# Cystatin C predicts cognitive decline in multiple system atrophy: A 1-year prospective cohort study

**DOI:** 10.3389/fnagi.2022.1069837

**Published:** 2022-11-28

**Authors:** Lingyu Zhang, Ruicen Li, Yanbing Hou, Bei Cao, Qianqian Wei, Ruwei Ou, Kuncheng Liu, Junyu Lin, Tianmi Yang, Yi Xiao, Wenxia Huang, Huifang Shang

**Affiliations:** ^1^Health Management Center, General Practice Medical Center, West China Hospital, Sichuan University, Chengdu, China; ^2^Department of Neurology, Laboratory of Neurodegenerative Disorders, Rare Diseases Center, West China Hospital, Sichuan University, Chengdu, China

**Keywords:** multiple system atrophy, cystatin C, cognitive decline, prospective study, movement disorder

## Abstract

**Background:**

Accumulating evidence has suggested that cystatin C is associated with cognitive impairment in patients with neurodegenerative diseases. However, the association between cystatin C and cognitive decline in patients with multiple system atrophy (MSA) remains largely unknown.

**Objectives:**

The objective was to determine whether cystatin C was independently associated with cognitive decline in patients with early-stage MSA.

**Methods:**

Patients with MSA underwent evaluation at baseline and the 1-year follow-up. Cognitive function was evaluated with Montreal cognitive assessment (MoCA). Changes in the MoCA score and the absolute MoCA score at the 1-year assessment were considered the main cognitive outcome. The cystatin C concentrations in patients with MSA and age, sex, and body mass index matched-healthy controls (HCs) were measured. A multiple linear regression model was used to test the association between cystatin C and cognitive decline.

**Results:**

A total of 117 patients with MSA and 416 HCs were enrolled in the study. The cystatin C levels were significantly higher in patients with MSA than in HCs (*p* < 0.001). Cystatin C levels were negatively correlated with MoCA score at baseline and at 1-year follow-up. Multiple linear regression model adjusted for potential confounders showed that baseline cystatin C levels were significantly associated with the MoCA score (*p* = 0.004) or change in the MoCA score (*p* = 0.008) at 1-year follow-up.

**Conclusion:**

Our results suggested that cystatin C may serve as a potential biomarker of cognitive decline in patients with early-stage MSA.

## Introduction

Multiple system atrophy (MSA) is an adult-onset progressive neurodegenerative disease that manifests clinically with autonomic failure, parkinsonism, and a cerebellar syndrome in various combinations (Wenning et al., 2022). The cause of MSA is unknown, but it has been proven that glial cytoplasmic inclusions, neuronal loss, and astrogliosis predominantly involved in striatonigral and olivopontocerebellar structures (Koga and Dickson, 2018). However, the current treatment is not effective and cannot delay the progress of the disease. The prognosis is poor with a median survival time range from 6 to10 years (Low et al., 2015; Cao et al., 2018).

In recent years, an increasing number of studies have reported that non-motor symptoms in patients with MSA are prevalent (Schrag et al., 2010; Colosimo, 2011; Cao et al., 2015; Zhang et al., 2017, 2022b), such as depression, anxiety, fatigue, sleep problems, and cognition impairment, which seriously affected their quality of life and increased the burden on their families and society. Additionally, previous studies have reported that patients with MSA can suffer from mild single or multiple domains cognitive deficits (Stankovic et al., 2014; Koga et al., 2017). The cognitive function of patients with MSA declined with the progression of the disease (Zhang et al., 2022a). Therefore, to discover the potential factors that predict such cognitive decline in patients with MSA is of great significance.

Cystatin C is an endogenous cysteine proteinase inhibitor belonging to the type 2 cystatin superfamily and encoded by the CST3 gene, which is presented in all human body fluids (Mussap and Plebani, 2004). Accumulating evidence has suggested that cystatin C was significantly associated with cognitive impairment in older adults, as well as patients with neurodegenerative diseases (Cathcart et al., 2005; Chen et al., 2015; Cui et al., 2020; Nair et al., 2020; Yang et al., 2021). A large prospective cohort study indicated that higher level of cystatin C was associated with cognitive impairment in older Chinese adults (Cui et al., 2020). A meta-analysis suggested that cystatin C could be considered as a predictor for the risk of cognitive impairment (Nair et al., 2020). Additionally, higher level of serum cystatin C was associated with worse cognitive performance in patients with Alzheimer’s disease (AD; Chen et al., 2021). The level of cystatin C was higher in patients with Parkinson’s disease (PD) with mild cognitive impairment (Chen et al., 2015; Yang et al., 2021). Our recent study observed a significant cognitive decline in patients with MSA after a 1-year follow-up (Zhang et al., 2022a). Cystatin C was found to be associated with the disease severity in patients with MSA (Ye et al., 2021). However, the association between cystatin C and cognitive decline in patients with MSA remains largely unknown.

Thus, a 1-year prospective cohort study was performed to explore the association between cystatin C and cognitive function, as well as whether cystatin C could be a potential predictor of cognitive decline in patients with early-stage MSA.

## Materials and methods

### Study design and population

**Figure 1:**
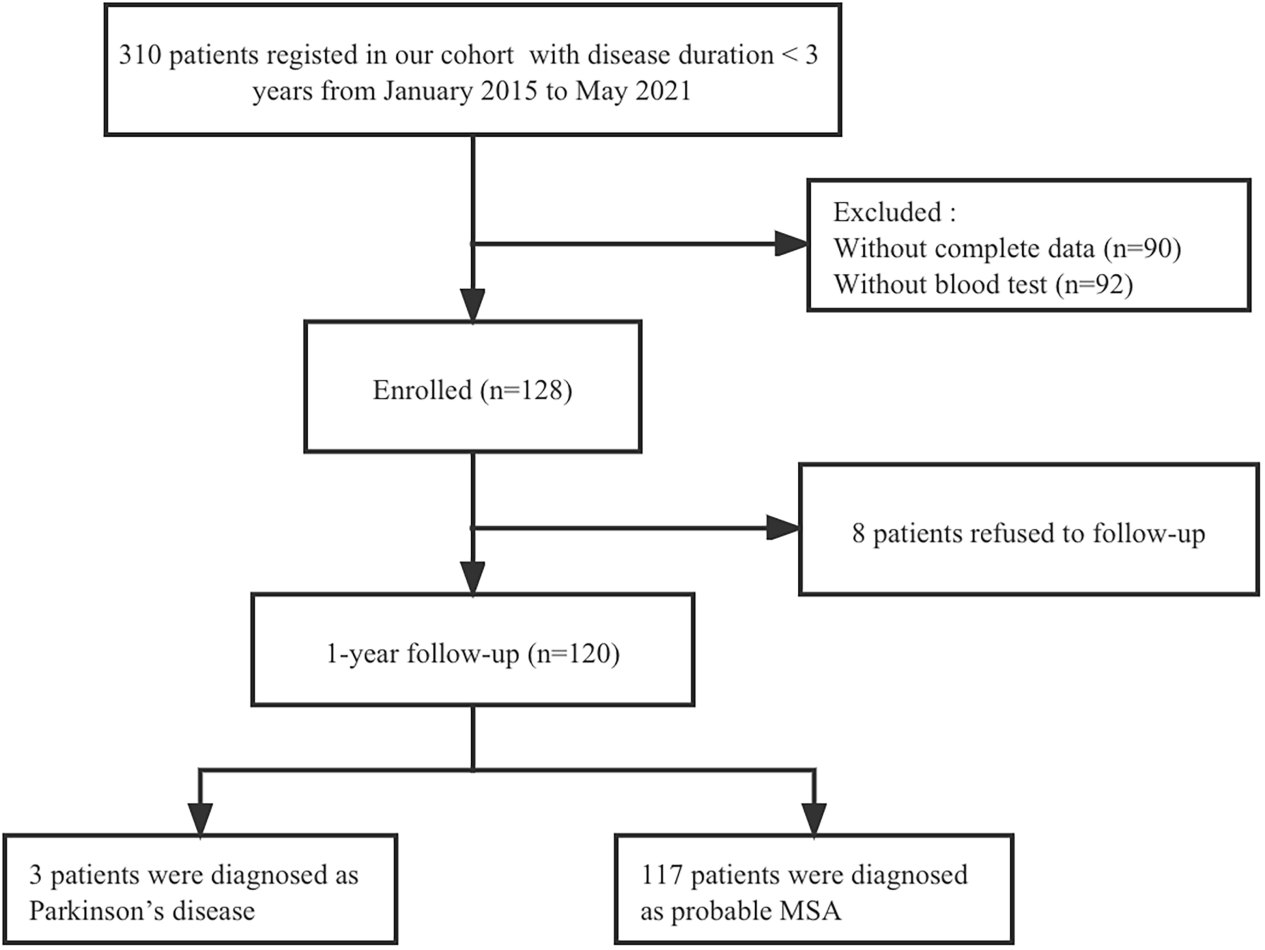
Study flow diagram.

This study recruited consecutive patients with MSA with a baseline disease duration of <3 years from the Department of Neurology, West China Hospital of Sichuan University, between January 2015 and May 2021. All patients underwent brain magnetic resonance imaging to exclude other neurological disorders. To exclude common forms of spinocerebellar ataxia (SCA), we performed screening for *SCA* genes including *SCA1*, *SCA2*, *SCA3*, *SCA6*, and *SCA7*. Patients underwent face-to-face interviews by neurologists at baseline and at 1-year follow-up. Among the 128 patients recruited at baseline, 8 patients refused to follow up and 120 patients completed the 1-year follow-up evaluation. Finally, 3 patients diagnosed with PD were excluded from the study, and 117 patients who met the criteria for probable diagnosis of MSA (Gilman et al., 2008) were included in the final analysis (Figure 1). A total of 416 healthy controls (HCs) were recruited from the Health Management Center, West China Hospital of Sichuan University, and their age, sex, and body mass index (BMI) were matched to the patients with MSA. HCs diagnosed with heart disease, hypertension, diabetes mellitus, or renal dysfunction were excluded. And none of them had any neurological diseases or psychiatric disorders.

### Hematological data collection

All participants underwent hematological examinations at baseline. Blood samples were taken by venipuncture in the morning after fasting from midnight. The following hematological tests were considered for this study: cystatin C, creatinine, urea, and estimated glomerular filtration rate (eGFR).

### Evaluation protocol

Baseline demographic and clinical data including age, sex, age of onset, height, weight, educational year, and disease duration was recorded. Disease duration was defined as the time between disease onset and evaluation. Disease onset was defined as the initial presentation of any motor symptoms (Parkinsonism or cerebellar dysfunction) or autonomic features, except erectile dysfunction. BMI was calculated as body weight (kg) divided by height squared (m^2^).

Patients with MSA underwent evaluation for disease severity and cognitive function at baseline and the 1-year follow-up. Disease severity was assessed using the Unified Multiple System Atrophy Rating Scale (UMSARS; Wenning et al., 2004), and the total UMSARS score was calculated as the sum of UMSARS parts I and II. Orthostatic hypotension (OH) was defined as a reduction in the systolic blood pressure by at least 20 mmHg and/or diastolic blood pressure by at least 10 mmHg after 3 min of standing up from a previous recumbent position of 3 min. Cognitive function was evaluated using a comprehensive and standardized cognitive screening tool [Montreal cognitive assessment (MoCA); Nasreddine et al., 2005]. The optimal cutoff scores of MoCA for cognitive impairment (CI) screening were 19 for individuals with no more than 6 years of education, 22 for individuals with 7–12 years of education, and 24 for individuals with more than 12 years of education (Chen et al., 2016).

### Statistical analysis

IBM SPSS software (version 26.0) was used for the statistical analysis. Continuous variables are exhibited as means and SD, and categorical variables as counts and percentages. Mann–Whitney U test was used to compare the continuous variables between different groups, since these data were non-normal distribution. Chi-squared test was used to compare the categorical variables between different groups. Spearman’s rank correlation coefficient was used to assess the correlation between baseline levels of cystatin C and clinical features at baseline and at 1-year follow-up. The change in the MoCA score at 1-year follow-up$=\frac{\left(\mathrm{MoCA score}\;\mathrm{at}\;1-\mathrm{year follow}\;\mathrm{up}-\mathrm{baseline MoCA score}\right)}{\left(\mathrm{date follow}\;\mathrm{up}\;\mathrm{in months}-\mathrm{date baseline in months}\right)/12}$, represents cognitive decline. A multiple linear regression model was performed to investigate the association between baseline cystatin C and the cognitive decline after adjusting for confounding factors, including age, sex, educational years, disease duration, diagnosis subtype, OH, eGFR, and baseline MoCA score. The MoCA score at the 1-year follow-up and change in the MoCA score at 1-year follow-up were used as separate dependent variables. Two-tailed *p* values <0.05 were considered statistically significant.

## Results

**Figure 2:**
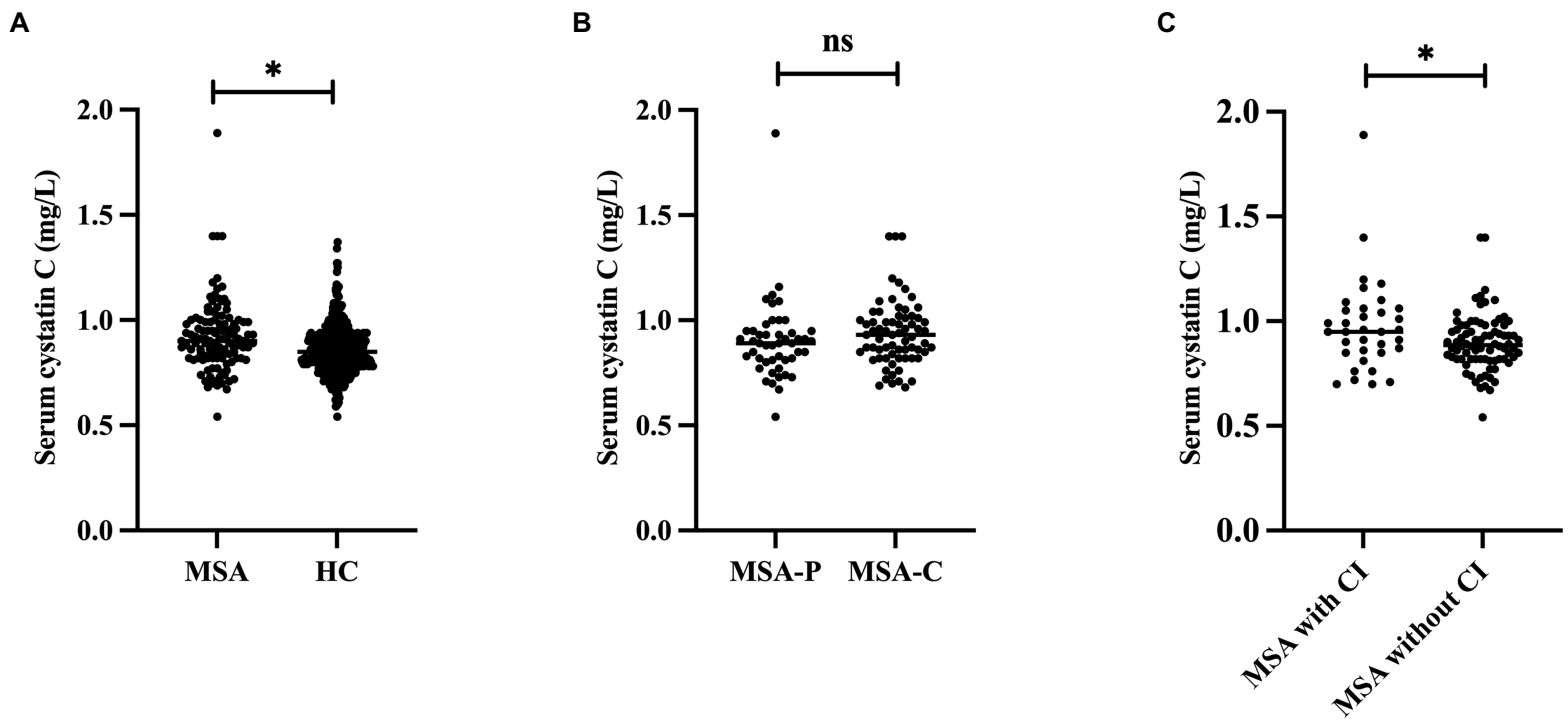
Comparison the cystatin C levels between different groups. **(A)** Comparison the cystatin C levels between MSA and HC. **(B)** Comparison the cystatin C levels between MSA-P and MSA-C. **(C)** Comparison the cystatin C levels between MSA with and without CI. *Significant difference.

Demographic and clinical information for MSA and HCs are shown in Table 1. A total of 117 patients with MSA and 416 HCs were enrolled in the study. The mean age of patients with MSA was 59.45 ± 7.50 years and the mean disease duration was 1.69 ± 0.74 years. There were no significant differences in the age, sex, or BMI between patients with MSA and HCs. The cystatin C levels were significantly higher in patients with MSA than that in HCs (0.92 ± 0.17 vs. 0.86 ± 0.12, *p* < 0.001; Figure 2A). There was no difference in cystatin C levels between MSA-P and MSA-C (0.90 ± 0.19 vs. 0.94 ± 0.16, *p* = 0.119; Figure 2B). The levels of cystatin C were significantly higher in MSA patients with CI than those without CI at baseline (0.98 ± 0.22 vs. 0.90 ± 0.14, *p* = 0.034; Figure 2C). A total of 29.9% (35/117) patients with MSA developed cognitive impairment (CI) at an early stage, while the frequency of CI was 50.4% (59/117) after 1-year follow-up. The levels of creatinine, urea, and eGFR exhibited that the renal function of patients with MSA was normal.

**Table 1:** Demographic and clinical information for MSA and HCs.

Variables	Time point	MSA (*n* = 117)	HCs (*n* = 416)	P^1^ value	P^2^ value
Age, year	baseline	59.45 ± 7.50	58.83 ± 6.47	0.495	-
Sex, *n* (%)	baseline				
Male		62 (53.0)	221 (53.1)	0.98	-
Female		55 (47.0)	195 (46.9)		
BMI	baseline	23.71 ± 3.88	23.95 ± 2.93	0.17	-
Diagnostic subtype, *n* (%)	baseline				
MSA-P		48 (41.0)	-	-	-
MSA-C		69 (59.0)	-		
Age of onset, year	baseline	57.76 ± 7.41	-	-	-
Disease duration, year	baseline	1.69 ± 0.74	-	-	-
Educational year	baseline	9.39 ± 3.63	-	-	-
UMSARS-I score	baseline	13.09 ± 5.78	-	-	<0.001*
	1-year FU	18.70 ± 7.12	-	-	
UMSARS-II score	baseline	14.68 ± 6.08	-	-	<0.001*
	1-year FU	21.87 ± 7.24	-	-	
UMSARS-IV score	baseline	1.78 ± 0.81	-	-	<0.001*
	1-year FU	2.38 ± 1.04	-	-	
Total UMSARS score	baseline	27.78 ± 10.98	-	-	<0.001*
	1-year FU	40.57 ± 13.71	-	-	
OH, *n* (%)	baseline	42 (35.9)	-	-	0.041*
	1-year FU	57 (48.7)	-	-	
MoCA score	baseline	22.66 ± 3.86	-	-	<0.001*
	1-year FU	20.13 ± 5.30	-	-	
CI, *n* (%)	baseline	35 (29.9)			0.001*
	1-year FU	59 (50.4)			
Cystatin C (mg/L)	baseline	0.92 ± 0.17	0.86 ± 0.12	<0.001*	-
Creatinine (umol/L)	baseline	67.31 ± 14.31	65.30 ± 13.60	0.302	-
Urea (mmol/L)	baseline	5.47 ± 1.49	5.05 ± 1.26	0.004*	-
eGFR (ml/min)	baseline	96.06 ± 12.94	96.49 ± 10.41	0.476	-

MSA: multiple system atrophy; HCs: healthy controls; BMI: body mass index; MSA-P: multiple system atrophy with predominant parkinsonism; MSA-C: multiple system atrophy with predominant cerebellar ataxia; UMSARS: Unified Multiple System Atrophy Rating Scale; OH: orthostatic hypotension; MoCA: Montreal cognitive assessment; CI: cognitive impairment; eGFR: estimated glomerular filtration rate; FU: follow-up. P^1^ value: comparison between MSA and HC. P^2^ value: comparison between baseline and 1-year FU. *Significant difference.

The UMSARS-I score, UMSARS-II score, UMSARS-IV score, and total UMSARS score increased significantly from baseline to 1-year follow-up in patients with MSA (*p* < 0.001). Meanwhile, the MoCA score of patients with MSA decreased significantly from baseline to 1-year follow-up (22.66 ± 3.86 vs. 20.13 ± 5.30, *p* < 0.001).

**Figure 3:**
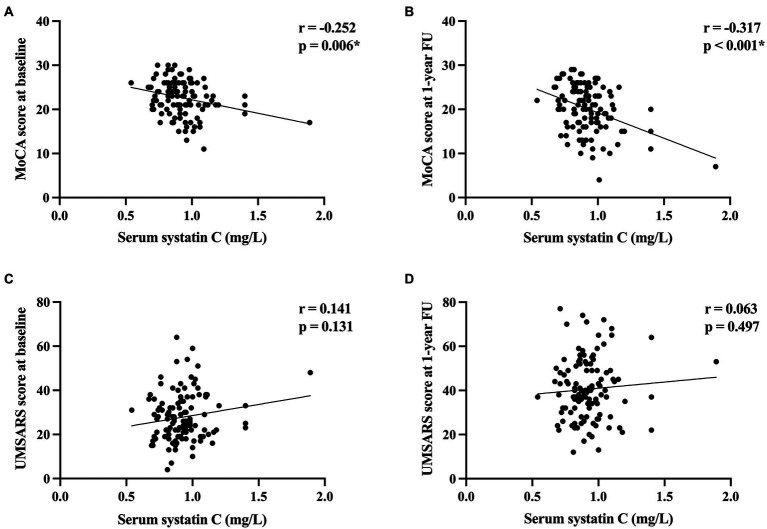
The correlation between cystatin C levels and cognitive function and disease severity in patients with MSA. **(A)** The correlation between cystatin C levels and MoCA score at baseline. **(B)** The correlation between cystatin C levels and MoCA score at 1-year follow-up. **(C)** The correlation between cystatin C levels and UMSARA score at baseline. **(D)** The correlation between cystatin C levels and UMSARS score at 1-year follow-up. *Significant difference.

Consequently, we then examined the correlation between cystatin C levels and clinical features in patients with MSA. Cystatin C levels were significantly negatively correlated with MoCA score at baseline and at 1-year follow-up (*r* = −0.252, *p* = 0.006; *r* = −0.317, *p* < 0.001, respectively; Figures 3A,B), while cystatin C levels were not significantly correlated with UMSARS score at baseline and at 1-year follow-up (*p* > 0.05; Figures 3C,D). We found that baseline cystatin C levels were negatively correlated with visuospatial and executive function domain (*r* = −0.188, *p* = 0.043) and naming domain (*r* = −0.264, *p* = 0.004) at baseline. And baseline cystatin C levels were negatively correlated with all cognitive domains of MoCA except language at 1-year follow-up (Supplementary Table 1). Multiple linear regression model adjusted for age, sex, educational years, disease duration, diagnosis subtype, OH, eGFR, and baseline MoCA score showed that baseline cystatin C levels were significantly associated with the MoCA score (β = −5.416, 95% CI −9.040 to −1.792, SE = 1.829, *p* = 0.004) or change in the MoCA score (β = −4.434, 95% CI −7.682 to −1.186, SE = 1.640, *p* = 0.008) at 1-year follow-up (Table 2).

**Table 2:** The correlation between baseline cystatin C and cognition outcome in patients with MSA in multiple linear regression model.

	Baseline cystatin C			
	β	95% CI	SE	*p* value^a^
MoCA score at 1-year FU	−5.416	−9.040 to −1.792	1.829	0.004*
1-year change in the MoCA score	−4.434	−7.682 to −1.186	1.640	0.008*

MSA: multiple system atrophy; MoCA: Montreal cognitive assessment; FU: follow-up.

a Adjusting for age, sex, educational years, disease duration, diagnosis subtype, OH, eGFR, and baseline MoCA score.

*Significant difference.

## Discussion

To our knowledge, this is the first study that examined the association between cystatin C and cognitive outcome in patients with MSA after a 1-year follow-up. The main finding demonstrated that cystatin C levels reflected cognitive functions in patients with early-stage MSA. We found a significant cognitive decline in patients with MSA after a 1-year follow-up. Cystatin C levels correlated with the cognitive status of patients with MSA. Additionally, in a prospective follow-up of these patients with MSA, we found that higher baseline cystatin C levels could predict a higher risk of cognitive decline after accounting for potential confounders, including age, sex, educational years, disease duration, diagnosis subtype, OH, eGFR, and baseline MoCA score. Therefore, these findings indicated that cystatin C may serve as a potential biomarker of cognitive decline in MSA.

Our finding that cystatin C levels were significantly higher in patients with MSA than in HCs, which was consistent with a previous case–control study in Chinese population (Ye et al., 2021). We observed a significant cognitive decline in patients with MSA after a 1-year follow-up, which was supported by our previous study (Zhang et al., 2022a). There was a negative correlation between cystatin C and cognitive status in patients with MSA. Additionally, multiple linear regression model showed that baseline cystatin C levels were significantly associated with cognitive decline in early-stage MSA after adjusting for age, sex, educational years, disease duration, diagnosis subtype, OH, eGFR, and baseline MoCA score. A meta-analysis including 12 studies with a total of 2,433 mild cognitive impairment patients and 1,034 controls suggested that cystatin C could be considered as a predictor for the risk of cognitive impairment (Nair et al., 2020). It has been reported that higher levels of cystatin C were associated with cognitive impairment in neurodegenerative diseases, including AD and PD (Chen et al., 2015, 2021; Yang et al., 2021). These findings may partially support our results since AD, PD, and MSA belong to neurodegenerative diseases, as well as PD and MSA belong to α-synucleinopathy.

Furthermore, chronic kidney disease is a recognized risk factor for cognitive impairment, which is usually judged by eGFR (Chu et al., 2022). The clearance of cystatin C through glomerular filtration can be affected by renal function. However, the increase of cystatin C levels in patients with MSA cannot be simply ascribed to the changes of renal function since the eGFR levels were not significantly different between patients with MSA and HCs. Additionally, cystatin C was associated with cognitive decline in patients with MSA at the 1-year follow-up independent of eGFR.

The pathogenesis of MSA is complex, but the propagation of misfolded α-synuclein from neurons to oligodendroglia and neuroinflammation caused by microglial activation play an important role (Jellinger, 2018). Cysteine cathepsins are essential in lysosomal degradation of α-synuclein (McGlinchey and Lee, 2015). Cystatin C is an endogenous cysteine proteinase inhibitor belonging to the type 2 cystatin superfamily, which is presented in all human body fluids (Mussap and Plebani, 2004). The high level of cystatin C can lead to accumulation of aggregated α-synuclein. And cystatin C also is a disease-associated protein involving in inflammatory and oxidative stress process (Xu et al., 2015). Additionally, cystatin C from neurotoxicant-injured rat dopaminergic neurons may be involved in the induction of microglial activation and exacerbation of neurotoxicity in a rat dopaminergic neuron-microglial cell-based *in vitro* model (Dutta et al., 2012). Cystatin C also can be released by mouse oligodendrocytes overexpressing human α-synuclein, triggering α-synuclein up-regulation and insoluble α-synuclein accumulation in a transgenic mouse mode of MSA (Suzuki et al., 2014). The neuropathological underpinnings of cognitive impairment in MSA are not clear. However, after a semiquantitative assessment of the burden of glial cytoplasmic inclusions and neuronal cytoplasmic inclusion (NCI) in autopsy-confirmed MSA, a greater burden of NCI in the limbic regions was found to be associated with cognitive impairment in MSA (Koga et al., 2017; Miki et al., 2020). Taken together, we hypothesized that cystatin C might be able to trigger NCI up-regulation accumulation in the limbic regions based on the above evidences. Further studies are needed to confirm our results.

The major advantages of the study include (1) we performed a prospective follow-up study; (2) we included patients with early-stage MSA; (3) we enrolled HCs. However, several limitations need to be addressed. First, we assessed cognitive function only with MoCA, a brief screening tool of global cognitive function. Detailed neuro- psychological tests can provide more comprehensive cognitive domains for assessing the correlation between cystatin C and cognitive domain declines in patients with MSA. And it will be considered in our further study. Second, the follow-up period was a relatively short since MSA is a rare and rapidly progressive condition. Third, all patients were diagnosed clinically without postmortem confirmation. Fourth, the correlation between cystatin C levels and disease severity was not significantly in our study, which may be because the patients enrolled in our study were at an early stage. Further large sample study with different disease duration and longer follow-up period are needed to clarify the issue.

## Conclusion

We found a significant cognitive decline in patients with MSA after a 1-year follow-up. Our results suggested that higher baseline cystatin C levels could predict a higher risk of cognitive decline after accounting for potential confounders. Therefore, these findings indicated that cystatin C may serve as a potential biomarker of cognitive decline in patients with MSA.

## Data availability statement

The original contributions presented in the study are included in the article/Supplementary material, further inquiries can be directed to the corresponding authors.

## Ethics statement

The studies involving human participants were reviewed and approved by Ethics Committee of West China Hospital of Sichuan University. The patients/participants provided their written informed consent to participate in this study.

## Author contributions

LZ and RL: research project: conception, organization, execution, design the statistical analysis, and wrote the first draft of the manuscript. YH, BC, QW, RO, KL, JL, TY, and YX: data collection. WH and HS: research project: conception, review and critique the statistical analysis and manuscript. All authors contributed to the article and approved the submitted version.

## Funding

The present study was supported by the funding of 1.3.5 project for disciplines of excellence–Clinical Research Incubation Project, West China Hospital, Sichuan University (Grant No. 2022HXFH023), Sichuan Science and Technology Program (Grant No. 2022ZDZX0023), and Sichuan Postdoctoral Program (Grant No. TB2022043).

## Conflict of interest

The authors declare that the research was conducted in the absence of any commercial or financial relationships that could be construed as a potential conflict of interest.

## Publisher’s note

All claims expressed in this article are solely those of the authors and do not necessarily represent those of their affiliated organizations, or those of the publisher, the editors and the reviewers. Any product that may be evaluated in this article, or claim that may be made by its manufacturer, is not guaranteed or endorsed by the publisher.
