# Supplementary material for: Cystatin C predicts cognitive decline in multiple system atrophy: A 1-year prospective cohort study
Source: Front Aging Neurosci. 2022 Nov 28;14:1069837. doi: 10.3389/fnagi.2022.1069837 (PMC9742413; doi:10.3389/fnagi.2022.1069837)
Supplement: Supplementary file 1 [file Table_1.docx]

Supplementary Table 1 The correlation between baseline levels of cystatin C and clinical features in MSA.

| Variables | Cystatin C | |
| --- | --- | --- |
|  | rs | p value |
| Age | 0.388 | <0.001* |
| Age of onset | 0.380 | <0.001* |
| Disease duration | 0.095 | 0.306 |
| UMSARS-I score at baseline | 0.163 | 0.079 |
| UMSARS-II score at baseline | 0.086 | 0.358 |
| UMSARS-IV score at baseline | 0.084 | 0.366 |
| UMSARS total score at baseline | 0.141 | 0.131 |
| MoCA score at baseline | -0.252 | 0.006* |
| Visuospatial and executive function | -0.188 | 0.043* |
| Naming | -0.264 | 0.004* |
| Attention | -0.162 | 0.080 |
| Language | -0.178 | 0.055 |
| Abstraction | -0.024 | 0.793 |
| Delay recall | -0.150 | 0.107 |
| Orientation | -0.099 | 0.288 |
| MoCA score at 1-year FU | -0.317 | <0.001* |
| Visuospatial and executive function | -0.270 | 0.003* |
| Naming | -0.198 | 0.032* |
| Attention | -0.263 | 0.004* |
| Language | -0.103 | 0.267 |
| Abstraction | -0.201 | 0.030* |
| Delay recall | -0.200 | 0.031* |
| Orientation | -0.200 | 0.031* |

MSA: multiple system atrophy; UMSARS: Unified Multiple System Atrophy Rating Scale; MoCA: Montreal cognitive assessment.

*Significant difference.
